# Implementing Digital Tools for Mental Health Support in Young Individuals in Colombia: Mixed Methods Feasibility Study

**DOI:** 10.2196/69749

**Published:** 2025-12-29

**Authors:** Laura Ospina-Pinillos, Débora L Shambo-Rodríguez, María Isabel Riaño-Fonseca, Mónica Natalí Sánchez Nítola, María Fernanda Ramírez-Castro, María Gabriela Calvo-Valderrama, Salvador Camacho, Carlos Gómez-Restrepo, Alvaro A Navarro-Mancilla, Ian B Hickie, Jo-An Occhipinti

**Affiliations:** 1 Department of Psychiatry and Mental Health, School of Medicine, Pontificia Universidad Javeriana, Carrera 7 # 40-62, 8th Floor, Building Hospital Universitario San Ignacio, Bogotá, 110231, Colombia, 57 3208320 ext 2705; 2 School of Psychology, Pontificia Universidad Javeriana, Bogotá, Colombia; 3 Swiss Tropical and Public Health Institute, Allschwil, Switzerland; 4 University of Basel, Basel, Switzerland; 5 Hospital Universitario San Ignacio, Bogotá, Colombia; 6 Keralty Medical Centers, Bogotá, Colombia; 7 Medicalnet SAS, Bogotá, Colombia; 8 University of Sydney, Brain and Mind Center, Sydney, Australia; 9 Computer Simulation & Advanced Research Technologies, Sydney, Australia

**Keywords:** mental health, young people, Colombia, eHealth, feasibility, usability, acceptability, Information and Communication Technologies

## Abstract

**Background:**

The growing prevalence of mental health disorders among young people is a pressing global concern, particularly in low- and middle-income countries where access to care is limited. Digital tools, leveraging Information and Communication Technologies, offer promising approaches to bridge these gaps.

**Objective:**

This study evaluated the feasibility of 2 digital mental health tools—Youth Collective Minds (YMC), a web-based platform, and Mental Beat (MB; Avicenna Research), a smartphone app—targeted at young individuals aged 18‐25 years in Bogotá, Colombia.

**Methods:**

Participants (N=35) engaged with both platforms over 3 weeks in this mixed methods feasibility study, which incorporated thematic analysis with a deductive framework for qualitative data. Univariate analyses were performed to examine baseline patterns and data distributions, while bivariate analyses were conducted to investigate relationships and associations between variables, providing a comprehensive evaluation of the platforms’ feasibility in the acceptability, demand, implementation, and practicality domains.

**Results:**

Participants were primarily women (22/35, 63%) with a median age of 23 (IQR 21-24) years. A total of 1308 annotations were coded: acceptability (annotations=707), demand (annotations=116), implementation (annotations=276), and practicality (annotations=209). Participants highlighted YMC’s psychoeducational resources and MB’s ease of use as strengths. However, technical issues, including server malfunctions and insufficient feedback, impacted engagement. Quantitatively, 83% (29/35) expressed willingness to reuse YMC and 83% (29/35) MB. Sensor data from MB indicated significant associations between psychological distress and smartphone usage. Participants with higher psychological distress showed greater median battery charging of 585 (IQR 321-615) compared to those without distress, 188 (IQR 42-309; *P*=.04). Poor sleep quality was also associated with increased median battery discharge of 2867 (IQR 1697.5-3935.5) compared to participants who reported sufficient sleep, 556 (IQR 200-2968; *P*=.003). GPS data showed that participants who visited more unique locations had lower psychological distress scores, with a negative correlation (*r*=−0.424; *P*=.05). In terms of platform usage, in YMC, surveys on emotions (30/35, 86%) and stress (28/35, 80%) were the most frequently completed, while telecounseling services were underused, with only 8.6% (3/35) of participants accessing mental health telecounseling. In MB, surveys of positive emotions (97.1%) and relationships (97.1%) were answered by more than 90% (32/35) of participants.

**Conclusions:**

This study demonstrated the feasibility and acceptability of digital tools for mental health support among Colombian youth, suggesting that these tools promote self-awareness and mental health management but require technical refinements to enhance engagement. The study’s limitations, including a small sample size and short duration, underscore the need for broader research. Implementing participant feedback, strengthening cybersecurity, and scaling these tools could address mental health challenges in low- and middle-income countries, where such interventions are critically needed. These digital platforms represent promising steps toward bridging gaps in mental health care access.

## Introduction

The growing prevalence of mental health (MH) disorders in young people has become a pressing global concern. According to the Global Burden of Disease (GBD) 2020, there was a 25% increase in the global prevalence of depression and anxiety disorders worldwide, and results suggested that a more pronounced effect was seen in those aged between 20 and 24 years [1,2]. In low- and middle-income countries (LMICs), major economic, social, and health care challenges exacerbate this already significant issue [2,3]. Colombia is an LMIC with a long history of armed conflict in which different factors, such as poverty, lack of access to quality education, stigma, and limited MH resources, among others, obstruct a proper addressing of the MH needs of its population and worsen the treatment gap [4,5]. Regarding the MH situation of youth in the country, the 2015 National Mental Health Survey (NMHS) suggested that the lifetime prevalence of any MH disorder for those between 12 and 17 and 18 to 44 years of age was 7.1% and 9.1%, respectively [6]. Suicides in the 18‐19 age group reach an alarming rate of 10.43 per 100,000 inhabitants, followed closely by the 20‐24 age group at 9.98 per 100,000, compared with a 6.16 per 100,000 rate for the overall population [7].

Young people are not routine users of traditional MH services [8], and due to their digital literacy, they tend to rely on the internet as an initial source of information and guidance for their MH concerns [9,10]. Evidence suggests that self-help interventions and resources, where the individual has no contact with a health professional, are effective in reducing depression and anxiety, increasing a sense of control, reducing helplessness, and improving overall MH [11,12]. Over the past decade, Colombia has experienced substantial growth in Information and Communication Technology (ICT) infrastructure, fueled by government programs such as Plan Vive Digital, initiatives from the Ministry of ICTs, and the rollout of 5G, as well as contributions from the private sector [13-16]. These initiatives have enhanced broadband access, upgraded telecommunications, and promoted digital innovation nationwide.

Given this increased use and access to digital technologies, ICTs are considered to have great potential as self-help tools in providing information, resources, support, and communication to youth, thereby increasing patient empowerment and participation [17-19].

Some ICTs, such as smartphones or smartwatches, can collect not only active data (real input from users) but also passive data from sensors in the devices that gather information on parameters such as geolocation, pedometer, and accelerometer, among many others. The information obtained allows researchers and clinicians to perform a “Digital Phenotyping” that can be used to infer individual behavior and characteristics and can potentially aid in the early detection of some MH disorders [20,21]. According to the World Economic Forum (2019), the latter, added to ICTs’ easy accessibility, is particularly interesting in LMIC settings, as they can help to overcome some of the MH barriers mentioned above [22]. Addressing this growing crisis requires a concerted effort to improve MH services, increase awareness, and provide support systems tailored to the unique needs of young people in LMICs [23-25].

Bogotá, the capital city of Colombia, presents a distinctive mix of opportunities and challenges for applying ICT in MH. Home to a predominantly young population [26], the city faces significant risk factors related to issues such as forced displacement, poverty, informal economies, violence, and inequality [27,28]. Despite these challenges, 94.1% of Bogotá’s inhabitants aged 5 years or older own a smartphone and over 80.6% have internet access [29]. These factors position Bogotá as an ideal setting for conducting a study using ICT tools for MH promotion and prevention.

Our study aims to evaluate the feasibility of using 2 digital MH tools in individuals aged 18 to 25 years in Bogotá, the capital city of Colombia. To the best of our knowledge, there is no available information regarding the use of other digital MH interventions targeted at the MH of young people in Bogotá.

## Methods

### Recruitment

Participants were recruited through convenience and snowball sampling methods from a database of individuals who agreed to be contacted for research purposes. Dissemination of flyers and digital pieces also occurred through institutional social networks. Given the nature of the study, we aimed for a target sample of 35 participants who fully complied with the study protocol. Eligible participants were required to be aged 18‐25 years, reside in the metropolitan region of Bogotá, have an Android smartphone with continuous internet access, and be native Spanish speakers. Exclusions included documented cognitive impairment and illiteracy. For enrollment, interested participants received study explanations and had any questions related to the study answered by researchers.

### Procedure

**Figure 1.**
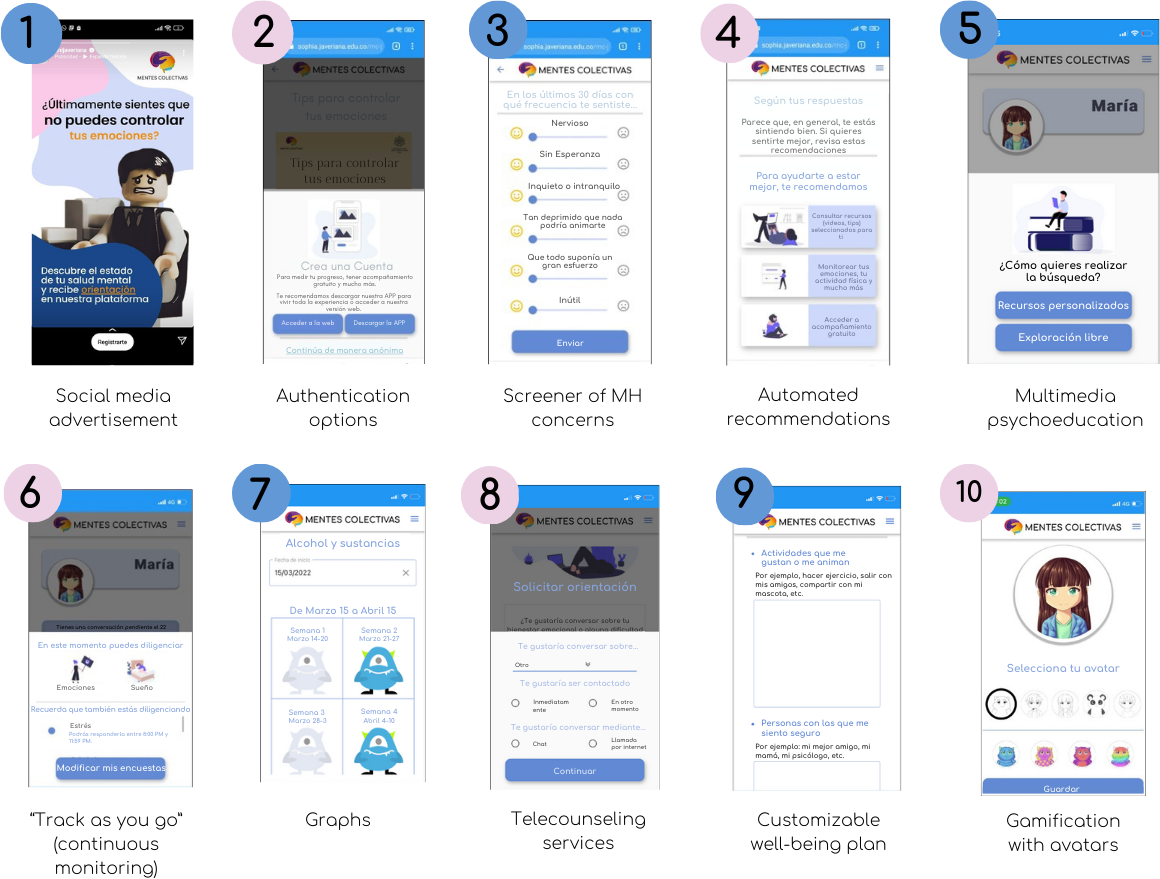
Main features of Youth Collective Minds, a web-based platform evaluated in a 3-week mixed methods feasibility study conducted among young individuals aged 18‐25 years in Bogotá, Colombia.

After enrollment, participants filled out a sociodemographic survey including self-reported gender identity (man, woman, transgender, and other) and were asked to use 2 MH digital tools for 3 weeks (refer to Figures1  2 for snapshots of their main features). Participants were not required to use both tools at the exact same time; however, they were encouraged to use them continuously during the study period. The first was Youth Collective Minds (YMC*; Mentes Colectivas para Jóvenes* in Spanish), a responsive website codesigned by the School of Medicine of the Pontificia Universidad Javeriana (PUJ), to promote self-management and MH help-seeking in youth through 7 features: a screener of concerns; automated, multimedia psychoeducation; “track as you go” (continuous monitoring); easy-to-understand graphs with legends; telecounseling services; customizable well-being plan; an SOS button for emergency services; and gamification with avatars [30]. A total of 13 surveys are included on topics related to MH, users freely choose their topics of interest, and YMC sends them 2 daily reminders to answer them. The tool has public access at all times, does not require downloading an app, and is available for youth older than 14 years of age or younger individuals with guardian consent.

**Figure 2.**
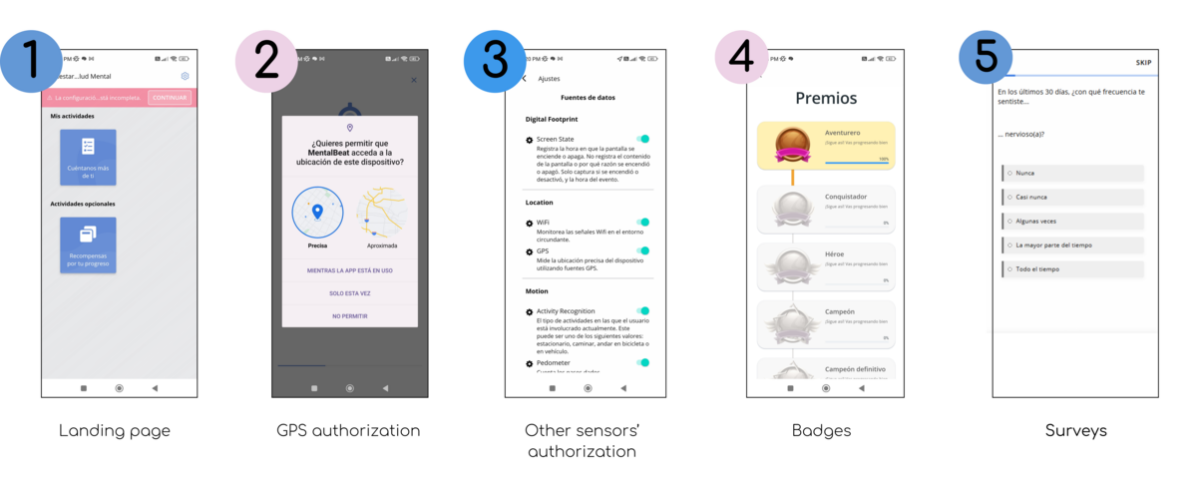
Main features of Mental Beat, a smartphone app tested alongside Youth Collective Minds in a 3-week mixed methods feasibility study with young individuals aged 18‐25 years in Bogotá, Colombia.

The second tool was Mental Beat (MB), which is based on the Avicenna Research app (Avicenna Research Inc). Avicenna is a participant-centric research platform designed to streamline the research process through seamless data collection, real-time monitoring and interventions, and enhanced participant engagement [31]. A 2020 cohort study in Australia used this app to prospectively follow several MH-related variables in more than 500 youth [32]. The study obtained good response rates for baseline questionnaires and acceptable response rates for follow-ups. Additionally, using both active and passive data, it was able to find significant associations, highlighting the potential of this adapted technology for MH monitoring. Based on this previous experience, we adapted the surveys for the Spanish-speaking Colombian context.

MB collects additional sociodemographic information, periodic questionnaires, and data from cell phone sensors, if the participant authorizes it. Each participant had to answer 1 or 2 surveys daily. The app has a gamification feature, in which users can level up and earn medals each time they complete a certain number of surveys. In the app, “leveling up” serves as a motivational tool to encourage continued user engagement and interaction by providing a sense of achievement and progress.

Participants were asked to log in to each platform at least once a day to complete the surveys and view the resources offered. If no activity was detected during the first week of the study, participants were contacted to have them comply with the study protocol, resolve any technical difficulties, or note reasons for withdrawal. Unreachable participants or those who voluntarily withdrew from the study were replaced to maintain the target sample.

The study used a mixed methods design to assess feasibility in 4 of the eight feasibility areas defined by Bowen et al [33]: (1) acceptability, which reflects how the target population received the tools including usability; (2) demand, evaluated through the usage of tools and their functionalities; (3) implementation, examined through the tools’ operation; and (4) practicality, assessed through users’ capacities and difficulties in using the tools. Although not part of Bowen and colleagues’ framework, we considered usability as a key aspect to evaluate the feasibility of an ICT and included it in the broader acceptability category as it relates to how easy a technology is to use, encompassing its effectiveness, efficiency, and user satisfaction.

### Data Collection

Three information sources were used: online semistructured interviews, online surveys, and platform data and analytics. Interviews, held online, included questions allowing assessment of a range of feasibility dimensions and were conducted by a senior psychologist and a psychologist specifically trained in the application of the interview guide (Multimedia Appendix 1), lasting between 45 minutes and 1 hour. The online survey included the Net Promoter Scale (NPS) [34], the System Usability Scale (SUS) [35], and questions about usage of platform functionalities. Finally, data were extracted from the platforms’ analytics. Table 1 explains in detail the methods used to measure each feasibility dimension.

**Table 1.** Feasibility dimensions and measurement methods applied in a mixed methods feasibility study of 2 digital mental health tools among young individuals aged 18‐25 years in Bogotá, Colombia.

Dimensions and instrument	Description
Acceptability	
Semistructured interview	Questions about general satisfaction, intent to continue use, satisfaction with monitoring features, perceived usefulness, perceived benefit of both tools, and opinions on the handling of personal data.
Net Promoter Score (NPS)	Willingness to promote the product measured by asking participants, “How likely are you to recommend [the platform] to a friend or family member?” on a scale from 0 (Not likely at all) to 10 (Extremely likely) [34]. Based on their responses, participants are categorized as promoters (9-10), passives (7-8), or detractors (0‐6). Next, the percentages of promoters, passives, and detractors are calculated. The final score is derived by subtracting the percentage of detractors from the percentage of promoters. This score can range from –100 (if every participant is a detractor) to +100 (if everyone is a promoter). A score above 0 indicates that most participants would recommend the platform, reflecting their satisfaction. Conversely, a score below 0 suggests that most users are dissatisfied and would not recommend it. In the industry, scores above 50 are considered to indicate good performance [36]. NPS is widely used in health care [37].
System Usability Scale (SUS) [35] (in its translated version into Spanish) [38]	Ease of use of the platforms, measured through ten 5-point Likert questions focusing on aspects of complexity, ease to learn, among others. Scores range from 0 to 100, with a widely used benchmark of 68 [38,39].
Online survey	Questions include intent to continue use (yes or no), perceived usefulness of the platforms in solving users’ concerns (rated from 0, Not useful at all, to 5, Very useful), and ratings of the platforms’ service (classified as bad, acceptable, outstanding, good, or very good).
Demand of use	
Self-reported platform use	Self-reported frequency of usage of various functionalities from MB^a^ (consulting digital medals) and YMC^b^ (PDF results download, sharing resources with family, friends or people close to them, using the well-being plan, searching for resources, and viewing the history of recommended resources).
YMC analytics	Number of clicks by a participant on the various functionalities of the platform (SOS button, well-being plan, Avatar, searching for resources, consulted resources, and telecounseling, among others).
Platform surveys	Answers to the monitoring surveys in YMC (well-being, stress, sleep problems, physical activity, self-esteem, bullying, relationships, loneliness, eating disorders, substance misuse, health perception, sexual health, and an emotions tracker) and MB (emotional distress, number of close ones, positive emotion, interpersonal relationships, physical activity, commitment engagement and achievement, study behavior, well-being, sleep, and eating behavior).
Sensors	Location, number of steps, screen time, and battery status.
Implementation	
Platform’s operation record	A record of the platform’s operation was maintained during the weeks of the study, to note any bugs or technical difficulties.
Dropout rate and reasons	Percentage of participants who withdraw from a study before its completion and reasons they provided for doing so.
Practicality	
Semistructured interview	Questions about users’ experience with the platforms, exploring what they considered motivating and demotivating factors, perceived conveniences and disadvantages, perceived positive and negative effects of usage, and difficulties encountered while using the platforms.

a MB: Mental Beat.

b YMC: Youth Collective Minds.

### Analysis

#### Quantitative Analysis

Descriptive analyses were conducted to determine whether the tools achieved benchmark values on NPS and SUS (acceptability dimension), as well as to assess platform usage (demand dimension). Inferential analyses were run as a means to explore the potential of these tools for inferring individual behavior and thus for supporting early detection and treatment response in MH.

For continuous variables, a normality test was conducted. Considering the sample size, medians and IQRs were preferred as measures of central tendency. For nominal variables, absolute and relative frequencies were described. For ordinal variables, absolute frequencies, relative frequencies, and medians were calculated.

The statistical analysis was conducted using R software version 4.3.2 (The R Foundation for Statistical Computing). We performed the Shapiro-Wilk test (*P>*.05 for normality), calculated central tendency measures, skewness, and kurtosis. Additionally, a bivariate analysis was carried out, with qualitative interpretations of the K6 scale and well-being scale as dependent variables. We used the chi-square test *(P<*.05) for associations among qualitative variables and both the Wilcoxon test (*P*<.05) and Mann-Whitney *U* test (*P*<.05) for quantitative comparisons. Correlation matrices were also created for survey results from MB and YMC with sensor activation data, and significance was determined with *P*<.05. Additional correlation matrices were produced between MB and YMC variables, also with *P*<.05. This paper presents only the results that reached statistical significance.

#### Qualitative Analysis

Other feasibility dimensions (implementation and practicality), as well as information extracted from interviews related to acceptability and demand, were evaluated through qualitative data analysis. For qualitative data, a thematic analysis using a deductive framework was conducted. The process began with data familiarization through transcription and repeated readings. A research assistant transcribed the interviews and developed an initial set of codes. A senior psychologist with extensive experience in qualitative research reviewed these codes and organized the material according to the dimensions proposed by Bowen et al [33]—acceptability, demand, implementation, and practicality. The themes were subsequently reviewed for internal validity. Both psychologists collaborated on the final analysis and the writing of the results. NVivo version 14 (Lumivero) was used for qualitative data analysis.

### Ethical Considerations

The research protocol was approved by the Institutional Review Board of the PUJ (FM-CIE-0886‐23). Participants signed an informed consent form on the REDCap (Research Electronic Data Capture; Vanderbilt University) platform before proceeding to register on the platforms. All study data were deidentified prior to analysis. The documents were stored on secure servers provided by the PUJ and protected by password access. Those who completed the study received a voucher of COP 50,000 (approximately US $13) as a gesture of gratitude for their time and willingness to participate. Potential participants were notified of this as part of the enrollment procedures; however, only those who fully complied with the study protocol received the voucher.

## Results

### Participants

In total, 35 individuals participated in the study, the median age was 23 (IQR 21-24) years. Most of them identified themselves as women (22/35, 63%) and the majority as heterosexual (30/35, 86%). In Colombia, socioeconomic status is classified into 6 tiers on an ordinal scale, with Tier 6 representing the highest level and Tier 1 the lowest. Tiers classify residential properties to differentiate public utility charges and allocate subsidies and contributions. While useful, tiers are not a direct classification of users’ payment capacity and they do not necessarily reflect other sociodemographic variables such as employment status or health coverage [40,41]. The majority of the sample belonged to the third and fourth socioeconomic tiers, with 54% (19/35) and 31% (11/35), respectively. Regarding educational level, 57% (20/35) had a university or technical degree. There were no people with disabilities, and none of them had been victims of the armed conflict (Table 2).

A total of 35 interviews were coded, adding up to 1308 annotations in the following codes: acceptability (annotations=707), demand (annotations=116), implementation (annotations=276), and practicality (annotations=209; Table 3).

**Table 2.** Sociodemographic characteristics of participants (N=35) in a mixed methods feasibility study of digital mental health tools for young individuals aged 18‐25 years in Bogotá, Colombia.

Characteristics	Participants
Age (years), median (IQR)	23 (21-24)
Age (years), n (%)	
18‐20	8 (23)
21‐23	16 (46)
24‐25	11 (31)
Gender, n (%)	
Woman	22 (63)
Man	13 (37)
Nationality, n (%)	
Colombian	35 (100)
Sexual orientation, n (%)	
Heterosexual	30 (86)
Homosexual	1 (3)
Bisexual	4 (11)
Socioeconomic tiers, n (%)	
3	19 (54)
4	11 (31)
5	3 (9)
6	1 (3)
Not stratified or no tier reported	1 (3)
Educational level, n (%)	
High school (between 6th and 12th grade)	15 (43)
College or university	15 (43)
Technical or technological	5 (14)
Belonging to ethnic groups, n (%)	
No	35 (100)
Disability condition, n (%)	
No	35 (100)
Victim of the armed conflict, n (%)	
No	35 (100)
Current psychiatric or psychological care, n (%)	
No	29 (83)
Yes	6 (17)
Psychiatric or psychological care in the past, n (%)	
No	9 (26)
Yes	26 (74)

**Table 3.** Number of coded annotations by feasibility dimension and platform in a mixed methods feasibility study with young individuals aged 18‐25 years in Bogotá, Colombia.

Dimension of feasibility	Youth Collective Minds (n=600), n (%)	Mental Beat (n=474), n (%)	Both platforms (n=234), n (%)	Total (n=1308), n (%)
Acceptability	349 (58)	229 (48)	129 (55)	707 (54)
Demand	35 (6)	33 (7)	48 (20)	116 (9)
Implementation	134 (22)	136 (29)	6 (3)	276 (21)
Practicality	82 (14)	76 (16)	51 (22)	209 (16)

### Acceptability

#### Youth Collective Minds

Based on survey responses, more than half of the participants considered the YMC service to be good (20/35, 57%), useful enough most frequently (20/35, 57%), and assured that they would use it again (29/35, 83%). These findings are consistent with interview results, for example, about half of the sample (16/35) is completely satisfied with the platform.

*Yes, I am quite satisfied with the platform. I feel that it was very useful to be able to analyze during the day: how I felt? how much I had been physically active or sitting? For example, when I was asked if I had been sitting, I started to analyze and I was like! Wow!, I’ve been sitting for a long time, even all the time, studying, in class... so it made me analyze more the perspective of everything I was doing during the day, which I didn’t do before using it*

When asked about what they liked most about the platform, the possibility of receiving personalized and easy-to-understand resources was frequently mentioned (23/35 participants).

*I really liked it a lot, I would say that I was very satisfied, I feel that the platform is very well designed, it is very pleasant [visually], it is not tedious, also the surveys are very well designed, not tangled at all. The resources that were available after each survey as well, I did not review all of them, but most of them. The poster on “the psychological first aid kit” stood out, I thought it was very cool, I took a screenshot, also the podcast, I listened to one on the topic of suicide and it really impacted me a lot.*

Filling out the MH surveys and seeing progress charts was considered useful by 26 of 35 participants. Additionally, 10 of 35 participants highlighted the telecounseling option as one of the most useful features of YMC, as they claimed that often they do not know where to seek help and having this option available made help-seeking easier and more accessible. Among these 10 participants, fewer than half (4/10) had no prior or current experience with psychiatric or psychological care. This suggests that their positive views on the telecounseling feature were likely independent of past service use. In general, the surveys were seen as relevant, easy to answer, short, and the possibility of choosing their topics was emphasized as a positive feature.

Usability is a key aspect to consider when studying a technology. Bowen and colleagues’ framework [33] for designing feasibility studies is not intended for technologies specifically, but for interventions more broadly. However, since the focus of this study is on 2 ICTs, usability measures were also included as part of the more general acceptability category. For YMC, participants gave this platform a score of 72.86 on the SUS, rendering it acceptable but requiring adjustments to improve its usability, mainly due to the technical difficulties that arose. Participants considered that these issues were the main cause of discouragement in using the platform. In interviews, dissatisfaction with the platform was primarily related to technical issues that limited access to the YMC web page for 3 days and generated difficulties logging in to their accounts. Nevertheless, a considerable percentage of participants (29/35, 83%) expressed the intention of using it again, and overall, they rated it as user-friendly, self-manageable, and free of contradictions. Participants considered that they could easily grasp how to use it without requiring any training or previous knowledge to start using it.

A score of 2.85 was obtained on the NPS scale: 34% (12/35) of the participants were promoters, 34% (12/35) passive, and 32% (11/35) as detractors. Despite this, the majority of participants (30/35) in the interviews expressed their intention to use the platform again, citing the personalized psychoeducational resources and the ability to track their MH through surveys as key factors for their decision.

*With YMC, I was super satisfied, I feel that it tried to keep track of all the things that were not well, such as mood, panic, stress, if I did physical activity. I really liked the website, not only because of that, but also because of the other options it provided, such as recommendations, my well-being plan. I found it to be very complete, I liked it very much*

#### Mental Beat

In surveys, this platform received ratings of good and very good from 49% (17/35) and 29% (10/35) of participants, respectively. In terms of usefulness, 25 of 35 participants considered it useful enough and 25 of 35 assured that they would use it again. When it came to overall satisfaction, 24 of 35 participants emphasized that the interface made it easy to use. Additionally, over a quarter of participants (16/35) appreciated the push notifications, which remained visible on their home screens and served as reminders to complete the surveys. Overall, participants found the app to function consistently well.

*With MB, I am very satisfied, the truth is that the application seemed to me quite robust, it did not get blocked at all, it was not intrusive at all, let’s say within the system, accessing the surveys was quite easy and in fact I found them a little bit more meaningful.*

Moreover, 23 of 35 participants found the MB app beneficial, with many highlighting the ability to monitor their MH as a key advantage. In addition, 18 of 35 interviewees noted that completing daily surveys helped them become more aware of their habits and MH.

*Let’s say that it emphasized things that one did not have in mind, or seemed very normal things, for example, regarding the quality of sleep, how much did you sleep? these are things that one began to identify, that one did not have in mind, so that’s why I thought it was pretty cool to manage the application.*

However, 20 of 35 participants expressed dissatisfaction with the app, citing the lack of feedback and resources after completing the surveys as a key issue. A lack of satisfaction that MB was only a data collection-focused, rather than MH care-focused app providing psychoeducational resources and other forms of support, led to a perception that the app became dull and monotonous over time. One participant stated:

*I am not very satisfied with MB because it seemed very basic to me, I don’t know, maybe it is because it is only a support of the virtual page of YMC, because I saw that in MB they only did surveys and that’s it, and I said: well, but what do they do with this information? is it then connected with the virtual platform, or is it only about filling out surveys and that’s it, so yes, I was left with the doubt and a bit of a lack of taste of MB.*

Considering the NPS, 40% (14/35) of individuals were promoters of MB, 29% (10/35) passive, and 31% (11/35) detractors. This resulted in an NPS score of 8.6 (SD 2.61). In addition to the above, 20 of 35 participants found the MB surveys quick and easy to answer and 12 of 35 participants found the app to work well overall.

Regarding the use of sensors, participants stated that they were not aware of their activation and tracking, even though they granted permissions when first downloading and using the app. In general, participants trusted the app, and the majority of the participants (26/30) had no concerns regarding the handling of their personal data or MH information. They considered that the data requested were not sensitive and mentioned that trust in the researchers and the institution made them feel secure with sharing their data. In general, participants felt that the data were protected, and they prioritized their well-being when using the tools over concerns about data management.

On the SUS, we obtained a score of 81.35, which implies that the platform was within the acceptable range and therefore was well received. Overall, 25 of 35 of the youth said that they would use the MB platform again, mainly because they felt that answering daily questions about aspects of their MH allowed them to become aware of their well-being and the changes they were experiencing. Ease of using the platform obtained the highest score of 4.6. Participants highlighted the easy access and use of the app and that they considered that questions could be answered in a short time and in a simple way. Regarding specific questions of the SUS, the lowest score of 3.91 was obtained for whether they would use this tool frequently. In interviews, the main reason cited by participants was the lack of feedback after answering the surveys and not being able to visualize progress during the week.

#### Demand of Use

We evaluated both demand and perceived demand. Regarding the first, the actual use of YMC and MB functionalities was assessed. In relation to YMC, the most used features were psychoeducational resources, which were used at least once by 100% (35/35) of participants, followed by avatars (27/35, 77%), and completing the well-being plan (18/35, 51%; Table 4). Notably, the least used feature was telecounseling used by 8.6% (3/35) of participants.

**Table 4.** Usage of Young Collective Minds features during a 3-week mixed methods feasibility study among young individuals aged 18‐25 years in Bogotá, Colombia.

YCM^a^ feature	Participants who used it (N=35), n (%)
Select an avatar	27 (77)
SOS button	8 (23)
Well-being plan	18 (51)
Telecounseling for sexual and reproductive health	1 (2.9)
Telecounseling for mental health	3 (8.6)
Mental health resources	35 (100)

a YCM: Youth Collective Minds.

During the 3 weeks of the study, each participant used MH resources (mean 18.48, SD 14.54; median 13, IQR 10-22.5).

Regarding the monitoring functionality of YMC, some surveys were programmed to be shown daily, weekly, or every 2 weeks (Table 5). Regarding daily surveys, they were all completed by at least half of participants. The most answered ones were emotions (30/35, 86%) and stress (28/35, 80%), and the least used was physical activity with 57% (20/35) of the sample answering it at least once. Considering the total number of responses, the most used daily surveys were sleep and emotions. For weekly surveys, the most used were self-esteem, relationships, and loneliness; for biweekly surveys, the total number of responses was similar for well-being and sexual and reproductive health. Among these, follow-ups were completed by the majority of participants for the emotions (26/35, 87%) and sleep (19/35, 83%) surveys.

**Table 5.** Usage of Young Collective Minds monitoring surveys during a 3-week mixed methods feasibility study among young individuals in Bogotá, Colombia.

YCM^a^ survey^b^	Number of times the survey was shown to participants^c^, n	Average number of responses (SD)	Participants who answered at least once (n=35), n (%)	Follow-ups, (n=35), n (%)	Total responses (n=695), n
Physical activity	21	5.05 (4.48)	20 (57)	16 (80)	101
Alcohol and substances	3	1.33 (0.52)	6 (17)	2 (33)	8
Nutrition	3	1.75 (0.71)	8 (23)	5 (63)	14
Self-esteem	3	1.64 (0.74)	14 (40)	7 (50)	23
Well-being	2	1.17 (0.41)	6 (17)	1 (17)	7
Bullying	3	1.50 (0.71)	2 (5.7)	1 (50)	3
Emotions	21	6.63 (4.36)	30 (86)	26 (87)	199
Stress	21	4.79 (3.92)	28 (80)	23 (82)	134
Health perception	3	1.60 (0.89)	5 (14)	2 (40)	8
Relationships	3	1.69 (0.75)	13 (37)	7 (54)	22
Sexual and reproductive health	1	1.00 (0)	6 (17)	N/A	6
Loneliness	3	1.69 (0.63)	13 (37)	8 (62)	22
Sleep	21	6.52 (4.45)	23 (66)	19 (83)	150

a YCM: Youth Collective Minds.

b Daily surveys were on physical activity, stress, sleep, and the emotions tracker. Biweekly surveys were the well-being and sexual and reproductive health surveys. All other surveys were programmed to be shown once a week.

c Number of times each survey appeared or was available to participants during the study period.

Regarding the MB surveys, all participants completed the surveys on demographic information, physical activity, and sleep at least once (Table 6). Surveys of positive emotions (97.1%) and relationships (97.1%) were answered by more than 90% (32/35) of participants. It is noteworthy that all participants (35/35, 100%) who responded to the physical activity survey answered it 2 or more times, showing better engagement with this survey topic on MB than on YMC. This survey was the most frequently presented to the participants.

**Table 6.** Usage of Mental Beat surveys during a 3-week mixed methods feasibility study among young individuals in Bogotá, Colombia.

MB^a^ surveys	Number of times the survey was shown to participants^b^, n	Average number of responses (SD)	Participants who answered at least once (n=35), n (%)	Follow-ups (n=35), n (%)	Total responses (n=848), n (%)
Close people	1	1.00 (0)	24 (69)	N/A^c^	24 (3)
Positive emotion	3	2.35 (0.77)	34 (97)	28 (82)	80 (9)
Relationships	3	2.26 (0.79)	34 (97)	27 (79)	77 (9)
Academic behavior	3	2.18 (0.81)	17 (49)	13 (76)	37 (4)
Well-being	1	1.00 (0)	17 (49)	N/A	17 (2)
Sleep	9	6.86 (2.03)	35 (100)	34 (97)	240 (28)
Physical activity	10	7.49 (2.23)	35 (100)	35 (100)	262 (31)
Tell us more about yourself (demographic)	1	1.00 (0)	35 (100)	N/A	35 (4)
Commitment, meaning, and achievement	3	2.45 (0.72)	31 (89)	27 (87)	76 (9)

a MB: Mental Beat.

b Number of times each survey appeared or was available to participants during the study period.

c N/A: not applicable.

Regarding sensors, MB logged various data types throughout the study, including the time of sensor activation and deactivation, duration, acceleration, and more. For this study, only the data described in Table 7 were used. Specifically, for activity type, MB uses a combination of sensors and algorithms to detect the most likely activity the user was performing. A detailed description of the data captured by each sensor is available on the Avicenna web page [42].

**Table 7.** Descriptive statistics of Mental Beat sensor data collected during a 3-week mixed methods feasibility study among young individuals in Bogotá, Colombia.

Sensor		Sensor description	n (%)	Median (IQR)
Screen status				
Off		Duration when the cell phone was off (minutes)	33 (94)	15502.88 (8143.75-23959.52)
On		Duration when the cell phone was on (minutes)	33 (94)	4321.55 (2419.1167-7829.4)
Pedometer		Number of steps	26 (74)	73078.5 (24735.25-91645.75)
Battery level		Battery level as a percentage (0%‐100%)	35 (100)	54.11 (30.82-67.75)
Battery status				
Charging		Frequency when the device was charging	35 (100)	199 (75-585)
Discharging		Frequency when the device was discharging	35 (100)	1739 (251-3500)
No charge		Frequency when no charge was detected	35 (100)	0
Full charge		Frequency when the device was fully charged	35 (100)	10 (0-127)
Activity type (AT)				
Stationary		Frequency when the AT was immobile	29 (83)	1713 (840-2981)
Inclination		Frequency when the AT was tilted	29 (83)	906 (248-1451)
On foot		Frequency when the AT was walking or running	29 (83)	195 (73-412)
On bicycle		Frequency when the AT was cycling	29 (83)	10 (4-23)
In vehicle		Frequency when the AT was in a vehicle	29 (83)	229 (26-642)

Specific to battery level and status, data from this sensor are relevant as they can serve as a measure of cellphone usage, which has been shown to be significantly associated with problematic internet usage in youth [43]. Other sensors, such as GPS, pedometer, and screen time, have been used previously in digital phenotyping research [43-45].

To assess demand for sensors, we evaluated the percentage of participants who had at least one record for each sensor, as well as the median value of the sensor (Table 7). Based on this approach, the most frequently used sensors were battery status and screen status (refer to Table 7 for sensor description), with data recorded for 100% (35/35) and 94.3% (33/35) of the sample, respectively. The activity type most frequently detected was the stationary state with a median of 1713 (IQR 840-2981) records, while the on-foot and on-bicycle states were recorded less frequently. The pedometer was active for 74% (26/35) of participants, providing step count data. Data on battery status showed that participants spent most of their time in a discharging state, while instances of full charge were infrequent.

The GPS sensor (Table 8) focused on travel and location metrics. Over the study period, the median total distance traveled by participants was 793.29 (IQR 140.43-1781.16) km, suggesting variability in mobility levels. The median total travel time recorded was 2501.13 (IQR 269.6875-3890.025) minutes, while participants made a median of 60056.5 (IQR 12725.5-77800.5) visits to different locations. In terms of unique places visited, the median was 42 (IQR 10-72), indicating diverse movement patterns among participants. Time at home had a median of 476.63 (IQR 11.7-927.7625) minutes, contrasting with a significantly higher median time spent outside the home at 1695.4 (IQR 174.1-3004.975) minutes, highlighting a tendency for more time in external environments.

**Table 8.** Descriptive statistics of GPS sensor data from Mental Beat sensors during a 3-week mixed methods feasibility study among young individuals in Bogotá, Colombia.

GPS sensors	Median (IQR)
Total distance (km)	793.285 (149.42-1695)
Total travel time (minutes)	2501.125 (269.6875 - 3890.025)
Total visits	50,022.5 (12725.5-77800.5)
Unique places	42 (10-72)
Time at home (minutes)	476.625 (476.625- 927.7625)
Time outside home (minutes)	1695.4 (174.1-3004.975)

These results showcase the demand for YMC and MB. During the duration of the study, usage was overall high; however, engagement varied according to the type of functionality, survey topic, or sensor.

Regarding perceived demand, 27 of 35 participants thought that other young people in Bogotá would use the platforms. Some (12/35), however, stated that they would need adjustments, including being in an app-based format (7/35), having a strong social media presence (1/35), and improving its interface (2/35). A group of participants (9/35) thought that only those already interested in MH topics would use them.

Given the high demand for MB and YMC, we also explored associations between sensors and MH-related measures. This allows us to explore the feasibility of not only engaging young people with digital health technologies for their MH, but also exploring their utility as potential indicators of MH state. The group with psychological distress had a mean battery level of 62.98 (SD 8.92), whereas the group without psychological distress had a mean battery level of 53.66 (SD 11.03). The results suggest that individuals with psychological distress had a significantly higher mean battery level compared to those without distress. This association was statistically significant, with a *P* value of .03, indicating that higher psychological stress correlates with a higher battery level. Previous research has linked problematic internet use with psychiatric symptoms [46]. This suggests that metrics like device usage for internet access, as well as related proxies such as screen time or battery level, could hold value for both clinical and research apps.

Likewise, a statistically significant association was found between battery charging status (indicating the phone is being charged) and psychological distress (*P*=.04). In individuals with psychological distress, the median number of battery charging records was 585 (IQR 321-615), while in those without psychological distress, the median was 188 (IQR 42-309). This finding suggests a relationship between higher levels of psychological distress and increased battery charging. Relatedly, Gansner and colleagues [43] found that a higher number of daily phone checks and phone sessions were related to lower scores in measures of problematic internet use and depression. Not in the direction found in previous studies, Gansner and colleagues proposed an alternative hypothesis: digital engagement may offer relief from negative affect in the short term. Similarly, in our sample, youth with psychological distress might be more prone to use their devices for short-term relief than youth without psychological distress, leading to more frequent charging. Interestingly, youth with psychological distress also exhibited higher battery levels, which could suggest they maintained these levels by frequently charging their phones.

Participants who perceived they slept enough had a median battery discharge of 556 (IQR 200-2968), while those who did not perceive they slept enough had a significantly higher median discharge of 2867 (IQR 1697.5-3935.5). This suggests a statistically significant relationship between battery discharge and perceived sleep quality *(P*=.003)*.* As for the activity type sensors, a statistically significant relationship was found between immobility and time spent in vehicles in relation to sleep quality (*P*=.03)*.* Participants who perceived they slept enough recorded a median of 1213 (IQR 218-2607) minutes of immobility, whereas those who did not perceive they slept enough reported a median of 2129 (IQR 1515-6701) minutes. This association was statistically significant.

Similarly, time spent in a vehicle also differed significantly between the two groups. Participants who perceived they slept enough had a median of 46 (IQR 17-525) recorded instances of being in a vehicle, compared to 262 (IQR 150-762) minutes for those who did not perceive they slept enough. This difference was statistically significant, with a *P* value of .05, suggesting that greater immobility is associated with a worse perception of sleep quality.

A Mann-Whitney *U* test was performed to compare Screen Status Off in the group that perceived they had not slept well and the group that perceived they had slept well. There was a significant difference in Screen Status Off between these two groups: z=−2.089; *P*=.04. This test indicates that participants who felt they didn’t sleep well had their phones off for a longer time.

There was a statistically significant negative correlation between the number of unique places visited by a person and their score on the K6 psychological distress test. For this analysis, a nonparametric Spearman correlation test was conducted, resulting in a correlation coefficient of −.424 (*P*=.05). This implies that the greater the number of unique places a person visited during the 3-week period, the lower their score on the psychological distress test. Unique places are defined as those locations that the participant visited only once during the study.

### Implementation

#### Overview

During the 3-week study, the YMC platform encountered multiple technical difficulties, including server malfunctions, web page errors (reported by 19/35 participants), slow registration processes, delays in starting sessions (22/35), and dissatisfaction with the lack of notifications (3/35). Other complaints included excessive emails, limited survey options, and issues visualizing results.

In contrast, the MB app had no major technical issues and was appreciated for its offline functionality, fast performance (18/35), and push notifications (8/35). However, some survey responses were not recorded or processed, affecting 3 participants, and inconsistent passive data recordings led to the exclusion of some data points. Both platforms faced complaints about rigid survey schedules, which often resulted in expired surveys. The screen state sensor occasionally recorded implausible durations exceeding 24 hours, resulting in 6 data points being excluded. Similarly, the activity type sensor, which categorizes activities (eg, walking and running), recorded discrepancies for one participant where “on-foot” activity data did not align with walking and running records, leading to the exclusion of that participant’s data. These issues highlight challenges in accurately capturing passive data for analysis.

Participant dropout involved 3 individuals. One completed the study but was unreachable for an interview, while the other two left due to technical issues with YMC, including unsaved responses and platform freezing.

#### Practicality

Participants reported several positive effects from using the platforms, including increased awareness of their MH and habits (21/35 for MB and 16/35 for YMC), with specific emphasis on sleep surveys helping them recognize the importance of sleep hygiene. Many participants noted that the platforms motivated them to adopt healthier routines (28/35 for MB and 25/35 for YMC) by encouraging mindfulness about daily activities and creating dedicated time for MH. In YMC, learning was frequently highlighted as a benefit (30/35), with participants gaining knowledge, validation, and support through resources, survey results, and graphs.

*When I get a sleep survey, or when I get a survey about screen time on the TV or apps, it makes me aware, like, 'Wait a minute! How much time did I spend on my phone today, or how much time did I spend lying down?' Or, for example, with questions like the one about physical activity—how much time did you spend being active or studying? [...] It’s like this awareness that shows you, 'Okay, there are things I can improve, there are things I can change,' and that’s all thanks to the app*

[MB]

However, participants expressed dissatisfaction with the lack of feedback in MB (18/35), suggesting it felt like a data collection tool without deeper engagement. A few participants (4/35 for MB and 1/35 for YMC) saw no positive effects, and boredom was noted as a potential barrier to long-term use (3/35 for MB and 4/35 for YMC). Stress from technical malfunctions (7/35 for YMC), frustration with forgetting surveys, and occasional device issues were also reported.

*It’s like you might get bored with the platform [YMC], I don’t know, but I do feel that. I also feel that it can kind of just stay there... Since it’s something you’re supposed to do regularly, like daily, I feel that within the routine and everything else, it can start to fade away and you might gradually set it aside.*

## Discussion

### Principal Results

Our feasibility trial evaluated the use of 2 digital MH tools, YMC and MB, among young individuals aged 18 to 25 years in Bogotá, Colombia—a setting characterized by limited MH resources despite increasing internet accessibility and extensive usage among youth (84% of individuals aged 12‐24 years) [23,47,48]. Both tools, previously described in the “Procedure” section, offer functionalities related to psychoeducation and mental health monitoring (passive and active data collection). The results demonstrated the feasibility of these tools in terms of acceptability, demand, implementation, and practicality, highlighting their potential to address MH needs in resource-constrained settings.

Emerging technologies have significant potential to enhance the accessibility and quality of MH care, making them valuable tools for both patients and MH professionals [49-52]. While the acceptability and use of these tools are well-established in high-income countries, their adoption in LMICs is still in its early stages [53]. Research and development in LMICs remain insufficient, and concerns persist regarding the scalability of such interventions in contexts where resources and connectivity are often limited [53-55]. However, the progressive growth of ICTs in LMICs presents an opportunity to address these challenges. In Colombia, despite widespread internet access and high usage among young populations [29,47], the availability of digital MH tools remains scarce due to factors such as costs, limited human resources, and infrastructure constraints [47,48]. Addressing these barriers through innovative and scalable solutions is essential for bridging the MH care gap in LMICs [56,57].

Our results were consistent with other researchers showing that participants were curious to explore their MH, open to trying these nontraditional tools, and their digital literacy allowed them to use them without requiring any prior training [58-61]. Overall, the tools were deemed acceptable by the majority of participants. Both were considered easy to use, and most expressed a willingness to use them again. In line with most research on digital tools, our participants stressed the importance of making the tools user-friendly and intuitive [62].

Despite their acceptance, technical difficulties and system bugs negatively impacted the user experience and reduced engagement for some participants [63,64]. Such issues in digital health tools can lead to high user attrition and, more critically, compromise patient safety [63,65]. Challenges such as app crashes, unresponsive designs, and inadequate error handling not only frustrate users but also disrupt health care quality [66]. A review by Kim et al [65] highlights that technical problems in health IT can hinder decision-making and delay care delivery, underscoring the importance of addressing these issues proactively. Aligned with this, our study suggests that rigorous testing, user-centered design, continuous monitoring, and the establishment of system failure protocols are essential to ensure the usability, safety, and reliability of digital health systems. These measures are critical to maintaining trust and achieving the intended outcomes in academic and clinical settings.

Through our experience, we found that young people are willing to share information about their MH through monitoring or sensors, as long as the process is straightforward and quick. This coincides with other findings that indicate high trust in digital tools among this population due to their digital literacy and familiarity with the digital environment [57,67,68]. Privacy and sharing information about their MH were not the main concerns for our participants, as seen in other studies [69]. In our case, most participants were not even aware of whether they had activated the sensors or not. This willingness and openness to try resources that might help them and provide information about their MH are what give digital tools significant potential with this population. However, this finding should be interpreted cautiously as participants were recruited from a pool of people who had already agreed to be contacted for participating in research, potentially introducing bias.

If this result is supported by further research, it would present a critical issue, as it makes young users particularly vulnerable by sharing sensitive information without proper assessment. This highlights the importance of creating and developing digital resources that protect their privacy and security, as they may not consider the information they share on these platforms as sensitive [70,71]. This underscores the need for digital tools to incorporate educational components that enhance users’ understanding of privacy rights, data use, and informed consent, rather than limiting participation to consent collection alone. Additionally, it underscores the responsibility of digital health providers to implement and maintain robust cybersecurity standards to safeguard health data. Given the highly sensitive nature of MH information, maximizing cybersecurity measures is essential to prevent unauthorized access, data breaches, or misuse, ensuring that user trust and safety are upheld [22]. Thus, while our results point to the importance of low-friction design, particularly for the monitoring or sensor components of digital tools, this should be balanced with robust safeguards to ensure user privacy and data protection. The use of sensors in modern devices is being explored as a tool to gather data on behavioral markers that may be linked to MH concerns [72,73]. While research on sensor-derived information is still emerging, it holds potential for providing valuable insights. Our findings showed interesting associations that allow for interpretation but do not support generalization or definitive conclusions. We found a significant association between psychological distress and mean battery level, where higher psychological stress correlated with higher battery level, as well as a correlation between increased battery discharge and higher levels of psychological distress. Another association was observed between perceived sleep quality and battery discharge: those who reported poor sleep quality had higher battery discharge, suggesting that individuals who spend more time on their devices tend to have poorer sleep quality. This suggests that individuals with increased psychological distress likely use their phones more (spend more time on them or use more apps), leading to faster battery drain, which may result in them charging their phones more frequently and having a higher average battery level. This finding is in line with previous research that suggests that excessive smartphone use is linked to psychiatric disorders, cognitive and emotional difficulties, sleep problems, and medical issues [74,75]. However, we are not proposing that smartphones are inherently harmful to youth MH; instead, this association warrants further exploration to better understand its implications.

The results also suggest that participants with lower sleep quality spent more time immobile and more time in vehicles. Supporting this finding, research shows that increased sedentary behavior is associated with poor sleep quality, with better sleep levels linked to lower adiposity and higher sedentary time correlating with sleep disturbances, regardless of physical activity levels [76,77]. Interestingly, in our sample, individuals who visited more unique locations (places visited only once during the study) had lower scores on the psychological distress test. It is well established that depression is associated with decreased activity, reduced motivation, and increased sedentary behavior [78]. Consistent with our findings, preliminary research suggests that certain GPS data patterns, such as reduced diversity in visited locations and spending more time in fewer places, are associated with more severe symptoms of depression.

Finally, our study shows that researchers interested in using data collection apps such as MB should consider incorporating responsive links to psychoeducational resources, feedback after survey completion, and data visualization. This approach can help sustain the engagement of young participants in long-term research studies and is consistent with previous studies highlighting the engagement benefits of including feedback in digital solutions [79,80].

### Limitations

This study has several limitations that should be considered when interpreting the findings. First, the small sample size of 35 participants and nonrandom sampling limit the generalizability of the results, as they may not fully represent the broader population of young people. Recruiting participants from a pool of people who had already agreed to participate in research could have also introduced bias. Their prior engagement and potential familiarity or trust in the university context could have influenced their openness toward digital tools and the research process, possibly leading to higher acceptability ratings than would be observed in less research-exposed populations. Additionally, the study’s short duration of 3 weeks may not provide sufficient insights into long-term usability, engagement, or the sustainability of the digital tools. The geographical focus on Bogotá further restricts the applicability of the findings, as the experiences of youth in other regions or countries with different cultural and socioeconomic contexts may vary significantly. Although Bogotá includes rural zones within its administrative boundaries, it is primarily an urban city, and only a small proportion of its population resides in rural areas [81]. Thus, while our sample reflects the urban characteristics of Bogotá’s youth, results from our study may not be generalizable to rural contexts where access to the internet and mobile technologies is more limited. Future studies should be carried out in other regions to explore how differences in connectivity, infrastructure, socioeconomic context, and digital literacy influence the feasibility of mental health tools. Finally, the restriction to participants aged 18‐25 years excludes other age groups, potentially overlooking usability and acceptability issues relevant to younger adolescents or older adults. These limitations highlight the need for further research to validate and expand upon these initial findings.

### Conclusions

The study highlights the feasibility of digital MH tools for young people in Bogotá, Colombia, with both YMC and MB demonstrating potential for fostering self-awareness and MH self-management. To the best of our knowledge, YMC is the only digital platform specifically targeting MH improvement in Colombia, with promising acceptance among youth. Incorporating participant feedback and refining features could further enhance its usability and impact.

Despite the potential, challenges such as technical issues, limited feedback, and a small, geographically specific sample size limit the generalizability of findings. Future research should expand to more diverse populations, explore long-term use, and address usability and cybersecurity concerns to build trust and scalability. These efforts could advance digital health tools as transformative solutions to bridge MH care gaps in Colombia and other LMICs.

## Supplementary material

10.2196/69749 Multimedia Appendix 1 Interview guide.

## Abbreviations

GBD: Global Burden of Disease
ICT: Information and Communication Technology
LMIC: low- or middle-income country
MB: Mental Beat
MH: mental health
NMHS: National Mental Health Survey
NPS: Net Promoter Scale
PUJ: Pontificia Universidad Javeriana
REDCap: Research Electronic Data Capture
SUS: System Usability Scale
YMC: Youth Collective Minds
