# Supplementary material for: Implementing Digital Tools for Mental Health Support in Young Individuals in Colombia: Mixed Methods Feasibility Study
Source: JMIR Form Res. 2025 Dec 29;9:e69749. doi: 10.2196/69749 (PMC12747665; doi:10.2196/69749)
Supplement: Multimedia Appendix 1 [file formative-v9-e69749-s001.docx]

**Semi-structured interview guide (translated version)**

Thank you for agreeing to participate in this interview. We are interested in learning about young people's experiences with the use of YMC and MB. To achieve this goal, in the next 45 minutes, we will review several questions about your experience with them, what you liked and disliked about them, and your suggestions for improvements, if any.

1. Did you use the YMC platform?

1a. During the 3 weeks of the study, how much did you use it?

2. Did you use the MB application?

2a. During the 3 weeks of the study, how much did you use it?

3. How satisfied are you overall with the YMC platform?

4. How satisfied are you overall with the MB application?

5. Would you use the YMC platform again? What motivates or discourages you from using it?

6. Would you use the MB application again? What motivates or discourages you from using it?

7. Was YMC useful to you? Which part of the platform was most useful to you?

8. Was MB useful to you? Which part of the application was most useful to you?

9. Do you think YMC and MB measured what they were supposed to measure? What surveys or sensors should be included?

10. If you were to need help with your mental health, do you think YMC and AMC would help you? Why?

11. Do you think young people in Bogotá would use YMC and MB? Why?

12. What advantages or benefits do you think the YMC platform offers?

13. What advantages or benefits do you think the MB application offers?

14. What difficulties did you encounter using the YMC platform?

15. What difficulties did you encounter using the MB application?

16. What surveys do you remember completing in YMC? How accurately were you describing your [INSERT surveys mentioned by the participant]?

17. What surveys do you remember completing in MB? What sensors do you remember enabling?

18. Are you concerned about your privacy or the security and handling of data collected by digital tools?

19. Do you think YMC was beneficial for you? In what ways?

20. Do you think MB was beneficial for you? In what ways?

21. Were there any negative consequences of using YMC or MB?

**Guía de entrevista semi-estructurada (original)**

Gracias por aceptar participar en esta entrevista. Nos interesa conocer las experiencias de los jóvenes con el uso de ABA y AMC. Para lograr este objetivo, en los siguientes 45 minutos, repasaremos varias preguntas sobre tu experiencia con ellas, las cosas que te gustaron de ellas y las que no y tus sugerencias para mejorarlas, si las hay.

1. ¿Utilizaste la plataforma ABA?

1a. Durante las 3 semanas del estudio, ¿qué tanto la utilizaste?

2. ¿Utilizaste el aplicativo AMC?

2a. Durante las 3 semanas del estudio, ¿qué tanto la utilizaste?

3. ¿Qué tan satisfecho estás en general con la plataforma ABA?

4. ¿Qué tan satisfecho estás en general con el aplicativo AMC?

5. ¿Utilizarías de nuevo la plataforma ABA? ¿Qué te motiva o desmotiva a usarla?

6. ¿Utilizarías de nuevo el aplicativo AMC? ¿Qué te motiva o desmotiva a usarla?

7. ¿ABA fue útil para ti? ¿Cuál parte de la plataforma fue más útil para ti?

8. ¿AMC fue útil para ti? ¿Cuál parte del aplicativo fue más útil para ti?

9. ¿Consideras que ABA y AMC medían lo que debían medir? ¿Qué encuestas o sensores se deberían incluir?

10. Si necesitaras ayuda para tu salud mental, ¿crees que ABA y AMC te ayudarían? ¿Por qué?

11. ¿Crees que los jóvenes de Bogotá usarían ABC y AMC? ¿Por qué?

12. ¿Qué ventajas o facilidades crees que tiene la plataforma ABA?

13. ¿Qué ventajas o facilidades crees que tiene el aplicativo AMC?

14. ¿Qué dificultades encontraste para utilizar la plataforma ABA?

15. ¿Qué dificultades encontraste para utilizar el aplicativo AMC?

16. ¿Qué encuestas recuerdas haber completado en ABA? ¿Qué tan precisa fue en describir tu [INSERTAR encuestas mencionadas por el participante]?

17. ¿Qué encuestas recuerdas haber completado en AMC? ¿Qué sensores recuerdas haber habilitado?

18. ¿Te preocupa tu privacidad o la seguridad y manejo de los datos recolectados por las herramientas digitales?

19. ¿Consideras que ABA fue beneficiosa para ti? ¿En qué sentido?

20. ¿Consideras que AMC fue beneficioso para ti? ¿En qué sentido?

21. ¿Hubo consecuencias negativas de usar ABA o AMC?
